# From High Strain Rates to Elevated Temperatures: Investigating Mixed-Mode Fracture Behaviour in a Polyurethane Adhesive

**DOI:** 10.3390/polym15122675

**Published:** 2023-06-14

**Authors:** Maria J. P. Ribas, Alireza Akhavan-Safar, Nicolas Pigray, Ricardo J. C. Carbas, Eduardo A. S. Marques, Catarina S. P. Borges, Sabine Wenig, Lucas F. M. da Silva

**Affiliations:** 1 Faculdade de Engenharia, Universidade do Porto, Rua Dr. Roberto Frias, 4200-465 Porto, Portugal; up201806409@edu.fe.up.pt (M.J.P.R.); nicolas.pigray16@gmail.com (N.P.); emarques@fe.up.pt (E.A.S.M.); lucas@fe.up.pt (L.F.M.d.S.); 2 Institute of Science and Innovation in Mechanical and Industrial Engineering (INEGI), Rua Dr. Roberto Frias, 4200-465 Porto, Portugal; cspborges@fe.up.pt; 3 Sika Automotive AG, Kreuzlingerstrasse 35, 8590 Romanshorn, Switzerland; wenig.sabine@ch.sika.com

**Keywords:** polyurethane adhesive, fracture energy, temperature, loading rate, mixed-mode

## Abstract

The investigation of the behaviour of adhesive joints under high strain rates is an active area of research, primarily due to the widespread use of adhesives in various industries, including automotive manufacturing. Understanding how adhesives perform when subjected to high strain rates is crucial for designing vehicle structures. Additionally, it is particularly important to comprehend the behaviour of adhesive joints when exposed to elevated temperatures. Therefore, this study aims to analyse the impact of strain rate and temperature on the mixed-mode fracture characteristics of a polyurethane adhesive. To achieve this, mixed-mode bending tests were conducted on test specimens. These specimens were subjected to three different strain rates (0.2 mm/min, 200 mm/min, and 6000 mm/min) and tested at temperatures ranging from −30 °C to 60 °C. The crack size was measured using a compliance-based method during the tests. For temperatures above *T_g_*, the maximum load supported by the specimen increased with an increasing loading rate. *G_I_* increased by a factor of 35 for an intermediate strain rate and 38 for a high strain rate from low temperature (−30 °C) to room temperature (23 °C). *G_II_* also increased for the same conditions by a factor of 25 and 95 times, respectively.

## 1. Introduction

Over the course of recent years, adhesive bonding has been undergoing continuous evolution and increasingly entering the industry more and more. In contrast to bonding methods such as riveting, which result in stress concentration zones adjacent to holes, and, welding, which complicates the joining process of dissimilar materials, adhesives enable a more advantageous and uniform distribution of the stress and production of lighter structures. Adhesives have gained widespread utilization across various industrial sectors, primarily due to their numerous advantages. In the automotive industry, specifically, polyurethanes have emerged as a favoured adhesive family. This preference is attributed to the exceptional properties and high bonding strength exhibited by polyurethane adhesives. They are commonly used to bond diverse materials for structural applications that are frequently exposed to conditions favourable to material degradation and bond deterioration, such as humidity, vibrations, and temperature fluctuations, among others. These conditions may deteriorate the joint strength as shown in some research studies [1].

Fracture energy is a key property of adhesives, particularly when it comes to joint design that follows the damage tolerance philosophy. Damage tolerance refers to the ability of a joint or structure to withstand and tolerate the presence of flaws or damage without experiencing catastrophic failure. In this context, fracture energy plays a critical role. It represents the energy required to propagate a crack through an adhesive joint, and it is a measure of the adhesive’s resistance to crack growth.

When designing joints based on the damage tolerance philosophy, it is essential to select adhesives with high fracture energy. Adhesives with a higher fracture energy can absorb more energy before crack propagation occurs, thereby providing greater resistance to failure. By incorporating adhesives with high fracture energy into joint designs, engineers can enhance the overall durability and reliability of structures, as these adhesives are better able to withstand the presence of cracks or flaws and prevent them from propagating further. The fracture of a component may occur under different loading conditions such as shear, tensile, and even mixed-mode loading, which is the most realistic scenario since the components are constantly under numerous stresses. Consequently, the fracture toughness of the polyurethane adhesive will be influenced by its surroundings, including the strain rate and temperature it experiences. This shows the importance of improving the design and manufacture of bonded structures, so it is possible to enhance their longevity and safety.

To address the challenges related to the conditions that affect the final performance of the joint, it becomes necessary to investigate how the material itself behaves under different loading rates and environmental conditions [2]. By improving the accuracy of fracture mechanism models, it is possible to predict the behaviour of bonded structures, optimize the performance of the structures, and develop more durable adhesives.

In recent years, there has been a growing interest in understanding the mechanical behaviour of adhesive joints under different loading rates and temperatures. Several studies have explored the impact of these variables on the strength and fracture behaviour of various adhesive materials, including crash-resistant epoxy and polyurethane adhesives. There are numerous studies that found many adhesives to be noticeably rate-dependent [3].

However, despite the number of recent studies, new trends have been set regarding strain rate and temperature effect on tensile, shear, and fracture properties. Concerning tensile/shear properties, Borges et al. [4], Jia et al. [5], and Machado et al. [6] revealed an incredibly dependent strain rate effect. Borges et al. [4] concluded that both tensile and shear strength increased with increasing loading rate. Although Jia et al. [5] found a similar type of behaviour at room temperature, the same was not so linear for other temperatures, where the stiffness of the adhesive even decreased while increasing loading rates at negative temperatures. In the study conducted by Machado et al. [6], for all displacement rates, the increase in temperature led to a decrease in tensile strength and the increase in strain rate led to an increase in tensile strength. Some authors verified if there could be any methodology that could influence the results of the tests in the study of fracture behaviour. Nunes et al. [7] studied a special test procedure where DCB tests were performed with the crack tip at a constant strain rate throughout the test, and it was revealed to be more consistent than tests performed at constant test speed; this is supported by the fact that the standard deviation of the test results decreased.

The strain rates experienced in an adhesive layer during testing using two commonly employed fracture toughness specimen configurations, DCB and ENF, were investigated by the same author. The primary goal was to comprehend the influence of adhesive properties on the progression of strain rate. The results demonstrated substantial variability in the strain rate along the bond line during DCB tests conducted at a consistent cross-head displacement rate. Specifically, the strain rate exhibited a threefold increase at the commencement of the test in comparison to its value at the end [8]. Regarding fracture behaviour, despite the small number of studies conducted, it remains an extensive unexplored field due to its conflicting results. Viana et al. [9] explored the strain rate dependence of a crash-resistant epoxy under low- and high-temperature conditions and found that the energy absorbed by the specimens (SLJ) under quasi-static conditions showed a decreasing trend with temperature due to the decrease in adhesive yield stress with temperature. Something similar was found by Jia et al. [10,11], where in both cases, the critical fracture energy in question decreased with increasing loading rate; however, in mode I, the critical energy release rate decreased with the temperature drop for the quasi-static condition while *G_IIc_* decreased with increasing temperature for higher strain rates. This aspect, however, was not investigated in the study conducted by Borges et al. [12]. The aim of this study was to create a finite element model (cohesive zone model also reviewed by Tserpesa et al. [13] that accurately represents the mechanical behaviour of adhesives under mode I fracture conditions, while also taking into account the dependence of properties on strain rate. With increasing strain rates, the two adhesives studied showed an increase in ultimate stress and critical energy release rate.

Bidadi et al. [14] intended to carry out a study of the effects of loading rate more in-depth, verifying its influence on mixed-mode fracture behaviour in an epoxy resin material. According to the results, fracture loads and corresponding fracture resistance values for each mode-mixity were decreased significantly by increasing the loading rate. This was reasoned by the fact that there was a reduction in the crack tip fracture process zone size in higher loading rates. However, Borges et al. [4] did not verify that, in both adhesives under study, the energy release rate, for all mode-mixities, increased as a function of the loading rate.

Perez et al. [15] aimed to fill the research gap on the strain rate and temperature effects of polyurethane adhesives, with the results showing that increasing the loading rate significantly increased the maximum strength of the DCB specimens, with the greatest sensitivity observed at room temperature.

Although there have been numerous studies investigating the effects of temperature and strain rate on adhesive behaviour, the majority of these studies have focused on epoxy adhesives rather than polyurethanes. As a result, there is a noticeable research gap when it comes to understanding the behaviour of polyurethane adhesives under varying temperature and strain rate conditions. Specifically, there is a lack of research on how strain rate and temperature interact with each other in mixed-mode conditions for polyurethane adhesives. Mixed-mode fracture, which involves a combination of different loading modes, has received less attention from researchers when considering various strain rates. This limited focus on mixed-mode fracture and strain rate variations in polyurethane adhesives means that there is still much to learn about their behaviour and response under different loading conditions. Taking this into account, the main aim of this work is to fulfil this research gap and understand the fracture behaviour of this polyurethane adhesive under different loading rates and temperatures. To do that, mixed-mode testing was performed for three different test speeds (quasi-static 0.2 mm/min, intermediate strain rate 200 mm/min, and high strain rate 6000 mm/min) and three different temperatures (low temperature −30 °C, room temperature at approximately 23 °C, and high temperature 60 °C).

## 2. Experimental Details

In this work, fracture envelopes of a polyurethane adhesive were determined at three different loading rates each at three different temperatures. Any subsequent mention with the nomenclature QS, ISR and HSR, will be relative to the quasi-static condition (0.2 mm/min), intermediate strain rate (200 mm/min), and high strain rate (6000 mm/min), respectively. A loading angle (mode-mixity) of 45° was implemented on a mixed-mode apparatus. However, at quasi-static mixed-mode conditions for room temperature, a mode-mixity of 60° was also adopted, for a more precise estimation of *G_IIc_* for quasi-static loading conditions. The experimental details are explained in the following sections.

### 2.1. Materials

DCB specimens were manufactured using high-strength steel (PM300) substrates. Throughout the course of this study, a ductile polyurethane-based adhesive with mechanical properties modified for industrial applications was used. The glass transition temperature, $T_{g}$, of the adhesive is −5 °C.

Detailed information regarding both the physical and mechanical properties of the adhesive is presented in Table 1.

### 2.2. Specimens’ Geometry and Manufacturing Procedure

**Figure 1:**
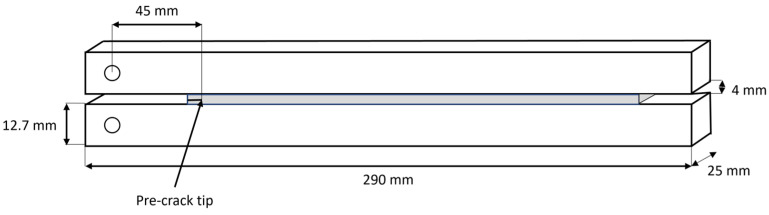
DCB specimen geometry.

Figure 1 illustrates the specimen geometry and respective adhesive thickness (4 mm).

The first step of the manufacturing procedure was to sandblast the substrate surfaces. This surface treatment is intended to enhance the adhesion of the adhesive to the surface by removing the iron oxides during the sandblasting process, creating a more adhesion-friendly surface and preventing the occurrence of interfacial failure [16,17,18].

The surface was then cleaned with acetone to remove any grease that might be on the surface. A primer (Primer-206, Sika, Switzerland) was then applied to improve the adhesion of the adhesive to the surface.

**Figure 2:**
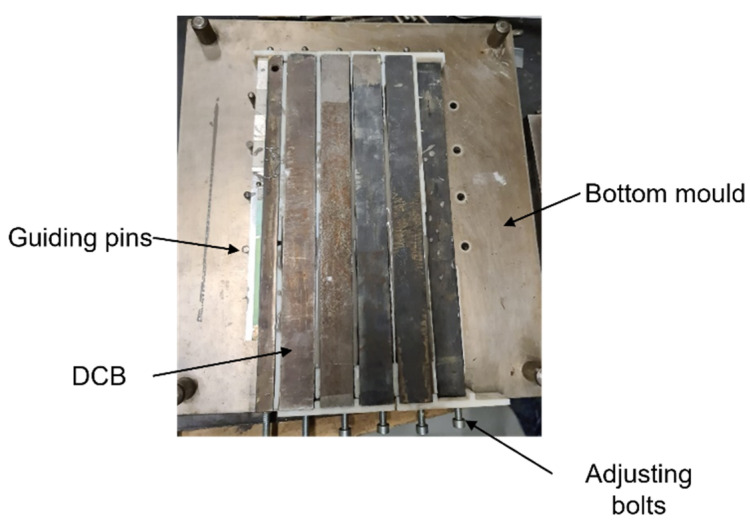
DCB specimens’ setup, only missing the top plate.

A typical mould for producing DCBs (Figure 2) typically comprises top and bottom plates with holes for the guide pins that hold the DCBs in place while the adhesive is being applied. However, to ensure that enough of the poured adhesive is retained within the joint during the curing cycle, the usual mould had to be modified by adding 3D-printed pieces due to the very low viscosity of the adhesive used in this study and the very thick bond line that was required. In order to guarantee a constant pre-crack size, a razor blade was used. This was positioned so that the initial crack had a length equivalent to 45 mm, as shown in Figure 1. This sheet was also sandwiched by two steel spacers in order to make a 4 mm thickness to position the crack in the middle of the adhesive layer. Besides this spacer responsible for positioning the razor blade, a 4 mm spacer was also inserted after the adhesive layer for better bond line thickness control.

After manufacturing, the curing process of the adhesive commences with an initial stage of 24 h at room temperature. Subsequently, a post-curing process ensues, involving an additional 4 h stage duration at 80 °C.

### 2.3. Test Methods

To perform mixed-mode fracture tests, several loading rates were taken into consideration. For each loading rate, the joints were tested at various temperatures, including low temperature, room temperature, and high temperature, always under mixed-mode settings. Table 2 summarizes the test conditions considered in this study.

All the tests were performed using an Instron 8801 (Norwood, MA, USA) servo-hydraulic testing machine equipped with a load cell rated to ±100 kN. A special, in-house-built thermal chamber was used to conduct experiments at various temperatures. To simulate the considered mixed-mode conditions, a mixed-mode apparatus was utilized. This equipment has previously undergone several validating standard fracture tests, confirming that the weight of the apparatus does not significantly affect the expected fracture energy [19]. To ensure substrate alignment during the curing cycle, a special mould was employed during the manufacturing process. Metal spacers were also calibrated to allow better control of the bond line thickness of the adhesive, which was 4 mm.

Different mode-mixities may be achieved for mixed-mode settings by adjusting the beam lengths ($s_{1}\;\mathrm{to}\;s_{4}$) in the mixed-mode apparatus. The forces exerted on the specimen’s top (*F*_1_) and bottom (*F*_2_) arms will alter as the beam length changes.

(1) $$F_{1}=F\times \frac{s_{1}}{s_{1}}$$

(2) $$F_{2}=F\times \frac{s_{1}s_{4}}{s_{3}\times \left(s_{3}+s_{4}\right)}$$

As a result, mode I and mode II are combined in the load delivered by the machine. *P_I_* and *P_II_* are established by

(3) $$P_{I}=\frac{F_{1}-F_{2}}{2}$$

(4) $$P_{II}=F_{1}+F_{2}$$

Additionally, the mode ratio (φ) is determined as below:

(5) $$\varphi_{apparatus}=\tan^{-1}\left\{\frac{\sqrt{3}\times \left(\frac{F_{1}}{F_{2}}+1\right)}{2\times \left(\frac{F_{1}}{F_{2}}-1\right)}\right\}$$

## 3. Data Reduction Approach

Accurate crack length measurement during fracture testing is challenging since the equipment design may limit visual monitoring and introduce additional measuring errors that may not be taken into account. During crack propagation, particularly in the case of the tested flexible polyurethane, a fracture process zone (FPZ) forms around the crack tip. This region experiences damage, leading to energy loss. When evaluating the length of the crack, it is important to consider the impacts of this FPZ and the energy dissipation caused by the damage. While using CBBM, a data reduction method based on linear elastic fracture mechanics, it is not necessary to monitor the crack length during testing, because an equivalent crack length is used instead of the real crack length. The compliance-based beam method, as the name says, is based only on the specimen compliance (C) from which the equivalent crack length can be estimated.

**Figure 3:**
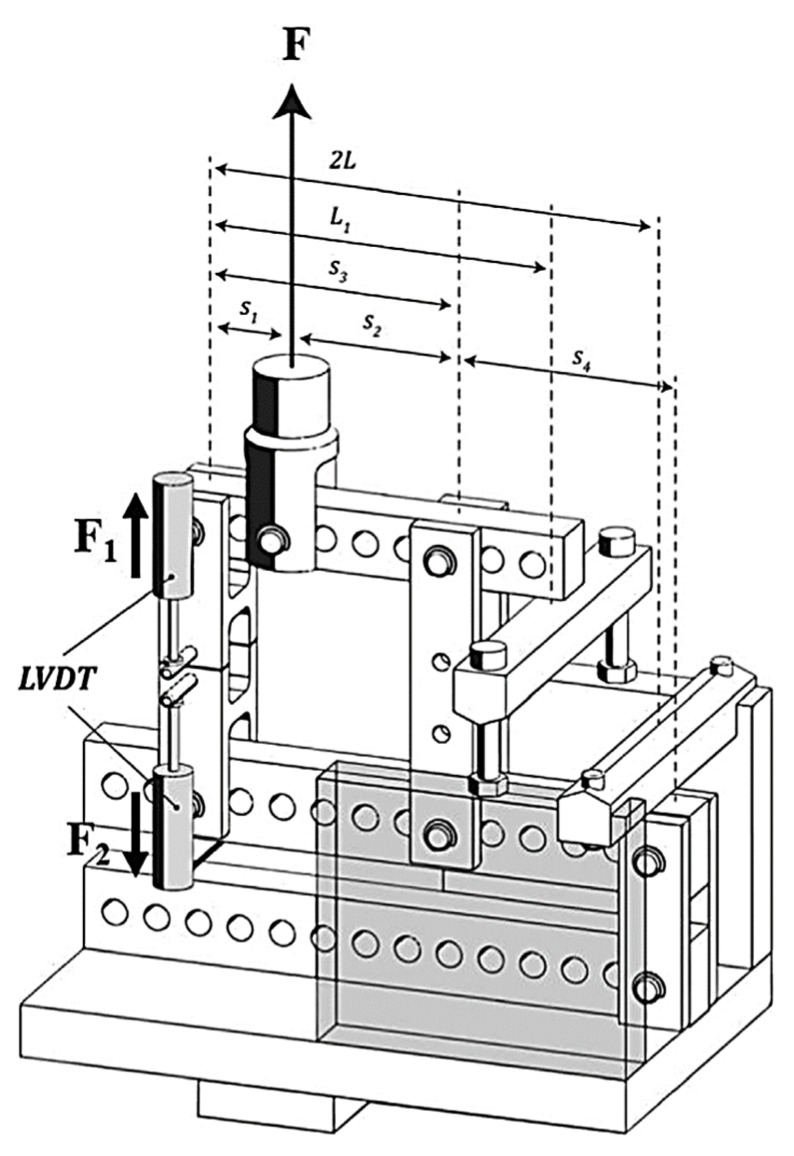
Schematic representation of the apparatus used for mixed-mode testing [19].

The fracture energy is divided into the pure mode I and mode II components for mixed mode, allowing a separate application of CBBM for each mode [20].

(6) $$C_{I}=\frac{8a^{3}}{EBh^{3}}+\frac{12a}{5BhG}$$

(7) $$C_{II}=\frac{3a^{3}+2L{L_{1}}^{2}}{2EBh^{3}}+\frac{6LL_{1}}{5BhG\left(2L-L_{1}\right)}$$

where *E* and *G* are the Young’s and shear moduli of the adhesive, respectively, and *a* is the crack length, *B* is the width of the joint, and *h* is the thickness of the specimen. *L* and *L*_1_ are defined in Figure 3. CBBM also considers the FPZ in the equivalent crack length ($a_{eq}$), which is possible to determine through the specimen’s compliance. For mode I, the formula is represented by Costa et al. [19]

(8) $$a_{eqI}=\frac{A}{6\alpha }-\frac{2\beta }{A}$$

knowing that *A* is calculated as below:

(9) $$A={\left(\alpha^{2}+108\alpha^{2}C_{I}+12\alpha^{2}\sqrt{\frac{3\left(4\beta +27C_{I}\alpha \right)}{\alpha }}\right)}^{\frac{1}{3}},\;\alpha =\frac{8}{Bh^{3}E_{f}},\;\beta =\frac{12}{5BhG}$$

For mode II, the formula is represented by the following equation [19]

(10) $$a_{eqII}={\left[\frac{2}{3}BEh^{3}\left(C_{II}-\frac{6LL_{1}}{5BhG\left(2L-L_{1}\right)}\right)-\frac{2}{3}LL_{1}\right]}^{1/3}$$

The fracture toughness values for each mode are then given by the following equations, considered the mode-mixity relations [19].

(11) $$G_{I}=\frac{9{P_{I}}^{2}}{B^{2}h}\left(\frac{{a_{eqI}}^{2}}{h^{2}E_{fI}}+\frac{1}{5G}\right)$$

(12) $$G_{II}=\frac{9{P_{II}}^{2}{a_{eqII}}^{2}}{16B^{2}E_{fII}h^{3}}$$

Figure 3 shows how to change the geometrical arrangements by modifying the distances designated as *s*_1_, *s*_2_, *s*_3_ and *s*_4_. The balance of forces is inevitably altered as the apparatus beam lengths are changed. As a result, distinct forces are applied to the upper adherend (*F*_1_) and lower adherend (*F*_2_). LVDTs were used to track the displacement of each substrate during the test.

**Figure 4:**
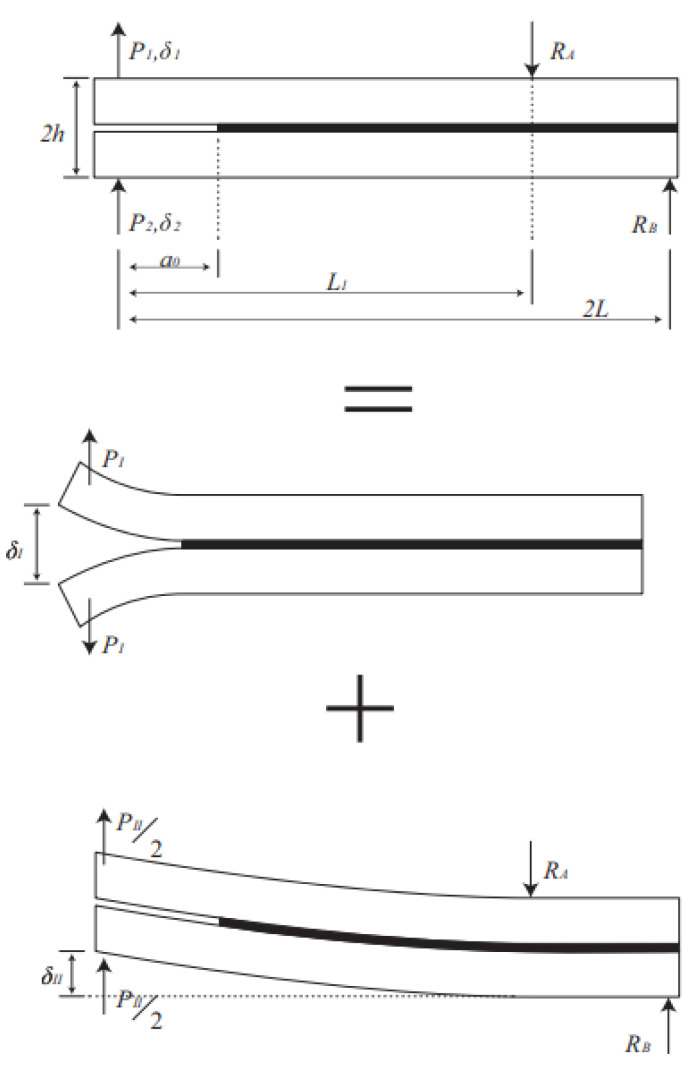
Mixed-mode loading conditions [19].

The fracture energy values for a mixed mode test are determined by taking the mixed mode loading as the sum of a mode I loading (DCB, opening mode) and a mode II loading (asymmetrical ENF, sliding mode), as illustrated in Figure 4.

## 4. Results and Discussion

In the following discussion, all graphs and results presented are relative to tests conducted at different temperatures and loading rates; however, all have the same mode-mixity set to 45°.

### 4.1. Joint Strength as a Function of Temperature and Loading Rate

**Figure 5:**
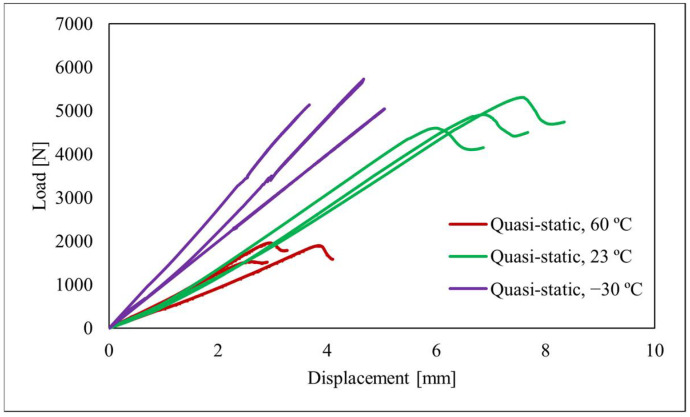
Load–displacement of the machine for test submitted to quasi-static conditions for a mixed-mode apparatus set up at 45°.

Figure 5 shows the load–displacement curves of the joints tested at different temperatures while maintaining the loading rate at 0.2 mm/min.

As shown in Figure 5, at −30 °C, the adhesive shows a stiffer behaviour as indicated by the slope of the curve. This indicates that at lower temperatures (below the *T_g_*), the adhesive is less flexible. This may happen as the molecular mobility decreases while transitioning from a rubbery state to a glassy state, which leads to a more compact polymer chain and subsequently a steeper slope, but it may still maintain a high load-bearing capacity as the room temperature results, due to a window that may exist where the adhesive does not undergo marked degradation of properties.

For temperatures above the *T_g_*, i.e., 23 °C and 60 °C, the results show a similar initial stiffness. However, at 23 °C, the displacement at failure is almost double compared to the results at 60 °C which means at higher temperatures, the adhesive experiences a degradation in its properties. Jingxin Na et al. [21] also found that the polyurethane adhesive in their study maintained ductility and increased resistance at low temperatures, which led to a higher joint strength, this was due to the fact that the adhesive had a relatively low *T_g_*. In the same study, it was found that the properties decreased with increasing temperature, such as the deformation to failure as found in the current study (Figure 5). Santos et al. [22] also discovered significant variations in the adhesive properties depending on the test temperatures for the tested polyurethane adhesive. A substantial difference was also observed between room temperature (23 °C) and 60 °C. Despite the adhesives exhibiting similar stiffness, the tensile elongation of the adhesive at room temperature (23 °C) was approximately twice as high as the results obtained at 60 °C. This finding inherently indicates a considerable degradation of fracture properties when the adhesive is subjected to elevated temperatures.

**Figure 6:**
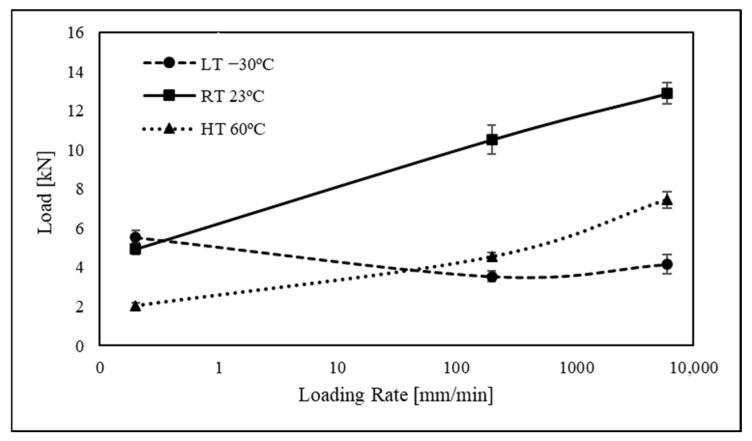
Maximum load of the machine as a function of loading rate and temperature for a mixed-mode apparatus set up at 45°.

Figure 6 shows the maximum load for different loading rates and temperatures. There is a clear change in the adhesive response accordingly with the temperature and loading rate applied. By increasing the test speed from a quasi-static condition to a high strain rate, the adhesive itself was able to withstand more load for temperatures above its $T_{g}$. This may be due to the fact that when the adhesive is at temperatures below its glass transition point, the adhesive becomes more brittle, with a more solid and rigid state. At this temperature, the adhesive is more likely to crack when submitted to stress/strain once it has less ability to dissipate energy through deformation. It is in this condition the adhesive is more sensitive to any types of defects/voids introduced during the manufacturing process.

However, in addition to the decrease in load-bearing capacity noticeable at temperatures below $T_{g}$, which suggests that the polyurethane adhesive is negatively affected by higher strain rates at this temperature, there is also a stabilisation of the maximum load after a loading rate of 200 mm/min. It may point to a modification in the way the polyurethane adhesive deforms or a range of strain rates where its mechanical properties are less sensitive. For the adhesive under investigation, it was observed that increasing the temperature from room temperature to high temperatures did not have a substantial effect on the rate of increase in maximum load concerning strain rate. This observation is supported by the similarity in the positive slope of the curves for both room-temperature and high-temperature conditions as shown in Figure 5.

### 4.2. Effects of Strain Rate on Mixed-Mode Fracture Energy

It should be kept in mind that fracture energy is essentially a function of the stress and displacement that the specimen has endured until failure. As a result, both factors need to be examined to fully understand how temperature and loading rate interact to affect fracture energy. The results show that a high loading rate has a significant impact on the fracture toughness of joints [23].

**Figure 7:**
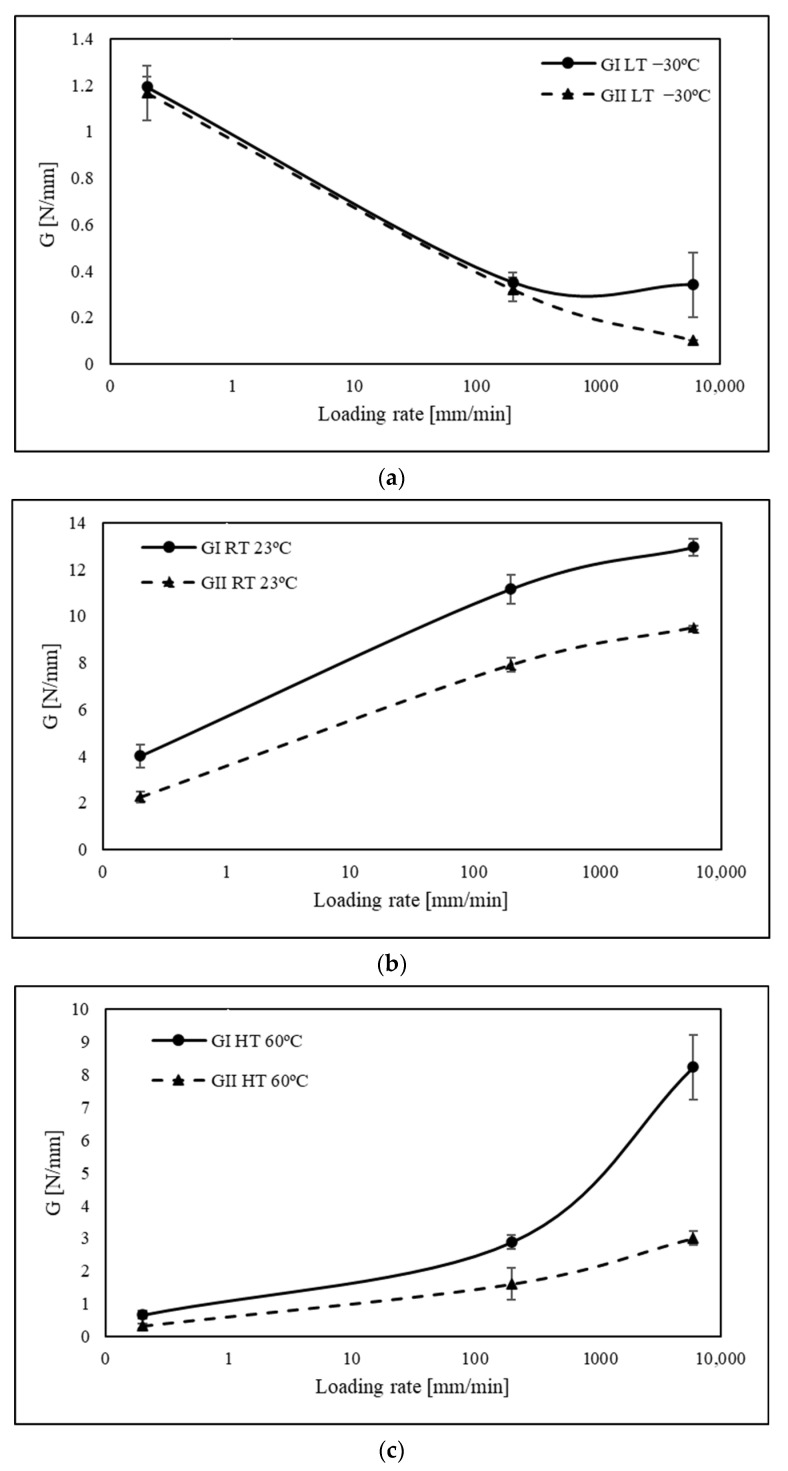
Critical fracture energy as a function of loading rate for different temperatures: (**a**) low temperature −30 °C; (**b**) room temperature 23 °C; and (**c**) high temperature 60 °C for a mixed-mode apparatus set up at 45°.

When analysing Figure 7 results, an abrupt decrease in critical fracture energy was detected for temperature below the $T_{g}$ for both the mode I and mode II components of mixed-mode fracture energy. It was also possible to observe a disparity between *G_I_* and *G_II_* for loading rate values higher than 200 mm/min at LT.

When the loading rate is lower than 200 mm/min, the fracture energy values are similar since the material can dissipate its energy through plastic deformation and microcrack growth in both fracture modes. However, with an increasing loading rate, the material starts to behave more rigidly and will have less time to dissipate energy, thus leading to a more brittle response. This results in a notable difference between mode I and mode II fracture since *G_II_* will decrease faster, thus being more sensitive to higher strain rates at low temperatures.

**Figure 8:**
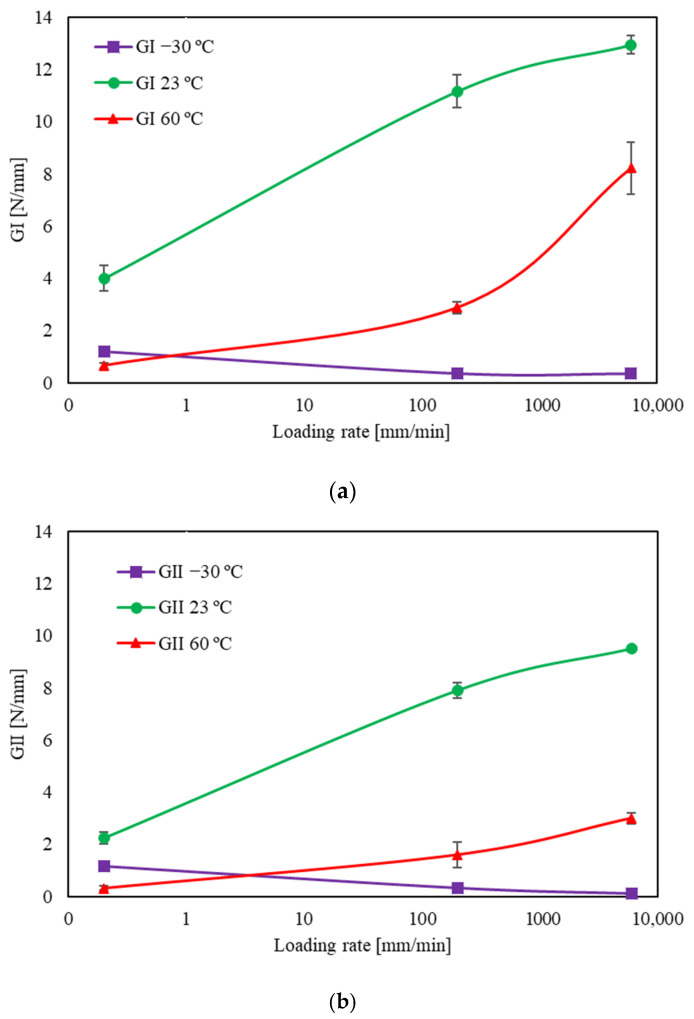
The effect of temperature on the fracture energy as a function of loading rate for different temperatures.: (**a**) Mode I part (**b**) Mode II part.

On the other hand, when tested above its glass transition temperature (23 °C and 60 °C), both fracture energies of the adhesive increased with the loading rate, except for the transition from 200 mm/min to 6000 mm/min for room temperature on the mode II part of the mixed mode. Once again, mode I and mode II reveal two different behaviours for high strain rates. During the test, the displacement rate is controlled and the 45° phase angle used on the mixed-mode equipment is set on the apparatus before testing. However, as the strain rate increases, there is a clear discrepancy between the *G_I_* and *G_II_* values, as previously mentioned. It would be expected that the strain rate would be the same for both modes, but mode I clearly grows at a faster rate, leading to the suggestion that the mode-mixity changes to pure mode I fracture behaviour. Bearing these aspects in mind, a comparative analysis of fracture energies was conducted, with a focus on the loading rate and each fracture mode. Based on Figure 8, it is evident that lower temperatures exhibit reduced sensitivity to strain rate. This phenomenon can be attributed to heightened sensitivity to strain rate resulting from temperatures surpassing the *T_g_*, wherein the responses for mode I and mode II fractures differ.

It should be noted that in the tests conducted, the displacement rate was controlled and not the strain rate. The displacement rate refers to the rate at which a material or specimen is displaced or deformed during a mechanical test, while the strain rate refers to the rate at which strain develops in a material or specimen during deformation. Despite the fact that the loading rate was set to a constant value for each specimen tested, the strain rate will exhibit distinct behaviours for mode I and mode II due to disparate sensitivities associated with each mode. Further experiments and numerical analysis should be carried out to have a better understanding of this phenomenon.

For the temperature of 60 °C, the fracture energy parameters show relatively similar behaviour for strain rates lower than 200 mm/min; however, for higher strain rates, *G_II_* increases about 10 times from the initial value in the quasi-static condition, while *G_I_* increases by a factor of 12.

This may happen because, at high temperatures, the polymer chains in the PU adhesive become more “mobile”, allowing a greater dissipation of energy and deformation before fracture and may increase *G_I_* and *G_II_*, but the increase is greater for *G_I_* because this mode is associated with the crack opening, and the separation of the bonded surfaces in mode I loading involves a more extensive displacement of the adhesive in the plane perpendicular to the crack, whereas *G_II_* is associated with crack sliding and shear deformation of the adhesive which involves a less extensive deformation in the plane of the crack; therefore, the increase in energy dissipation and high-temperature deformation has a greater effect on *G_I_* than *G_II_*.

With these results in mind, it can be seen that *G_I_* is slightly more sensitive to strain rate at high temperatures than mode II.

When comparing results at 23 °C and 60 °C, in general, it can be observed that fracture energy at room temperature (23 °C) is more sensitive to strain rate effects compared to the tests conducted at high temperature (60 °C). This finding is consistent with the results presented by Jun Zhang et al. [24], who investigated the behavior of silane-modified polyurethane sealant. Their study demonstrated that the material exhibited temperature dependence at a constant strain rate and rate dependence at room temperature. However, at high temperatures, the results showed lower sensitivity to the strain rate.

### 4.3. Temperature Effect

**Figure 9:**
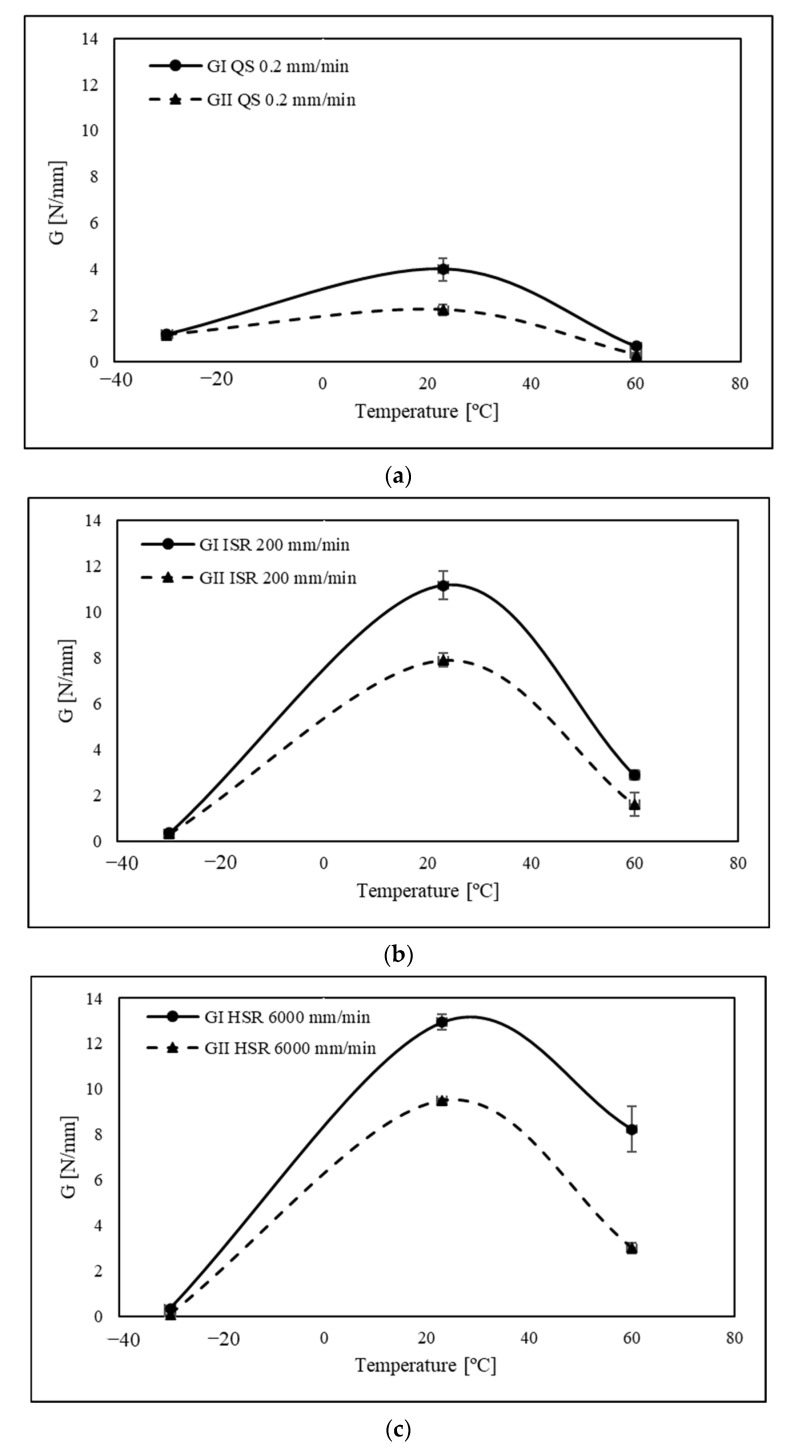
Critical fracture energy as a function of temperature for different loading rates for a mixed-mode apparatus set up at 45°: (**a**) critical fracture energy under quasi-static conditions, 0.2 mm/min (**b**) critical fracture energy under intermediate strain rate conditions, 200 mm/min (**c**) critical fracture energy under high strain rate conditions, 6000 mm/min.

Under quasi-static conditions, there was an increase of 3.4 times in fracture energy mode I from the temperature below $T_{g}$ to the temperature of the room, and this discrepancy was enhanced with an increasing loading rate. However, the same was not found for the other temperatures. In all those experiments, the critical fracture energy for both modes decreased when the temperature was above room temperature, as can be verified in Figure 9.

When an adhesive is exposed to a higher temperature than its $T_{g}$, it undergoes a reversible glass transition, in which the adhesive becomes softer and more viscoelastic. Yet, like in this case, when the adhesive is subjected to a very high temperature when compared with the glass transition temperature, thermal degradation may occur, which can lead to a permanent loss of its mechanical and adhesion properties [25].

**Figure 10:**
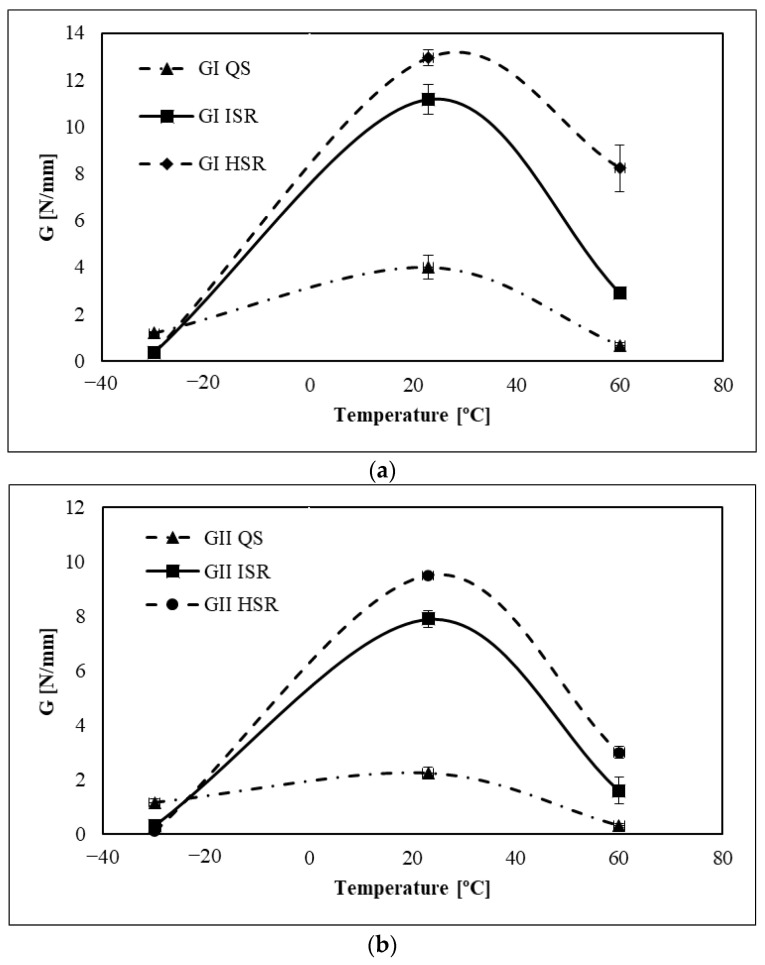
Fracture energy modes as a function of temperature: (**a**) Mode I part (**b**) Mode II part.

According to the results presented in Figure 10, between −30 °C and 23 °C, there was an increase in *G_I_* by a factor of 35 for intermediate strain rate and an increase by a factor of 38 for high strain rate. Regarding *G_II_*, fracture energy increased by a factor of 25 for the intermediate strain rate and by a factor of 95 for the high strain rate.

This clearly raises some questions about the sensitivity of *G_I_* and *G_II_* to strain rate and temperature. In the transition from ambient to lower temperatures, it is evident that mode I exhibits a significantly greater degree of sensitivity. Within the temperature range of 23 to 60 °C, under conditions of quasi-static loading and intermediate strain rates, mode I continues to show greater sensitivity. However, at high strain rates, the sensitivity of both modes appears to be comparable.

Comparing the ratio of *G_I_*/*G_II_* for intermediate (0.45) and high strain rate (2.74) at high temperature, it is clear that there is a large gap between these two values, which once again indicates the increasing discrepancy between *G_I_* and *G_II_* as the strain rate increases.

The best adhesive’s performance was obtained for higher loading rates at room temperature, and the adhesive itself showed a higher sensitivity to temperature than the loading rate. The specimen subjected to a high strain rate at low temperatures showed the worst performance.

Lopes et al. [26], while studying the temperature effect on an epoxy adhesive subjected to different mode-mixities, also concluded that the temperature sensitivity was more noticeable in tensile loading conditions when compared to shear mode and that the tensile fracture energy dropped with increasing temperature. Santos et al. [22] also found that an increase in temperature from 23 °C to 60 °C led to a decrease in strength and ductility for the adhesive in the study, which translated into a huge decrease in fracture toughness in mode I.

### 4.4. Fracture Envelope

The overall conclusion from published papers is that as the strain rate increases, structural adhesives’ fracture energy generally tends to decrease [10,11]. When exposed to impact stresses, an adhesive’s basic mechanical properties, such as tensile strength, may improve, but the adhesive becomes somewhat more brittle.

Considering the experimental results, in fact, the increase in loading rate, for the same temperature, leads to a reduction in the adhesives’ fracture energy for temperatures below its $T_{g}$. Yet, the same is not true for tests performed at temperatures above the glass transition temperature, namely, for room temperature and 60 °C. As previously mentioned, this may be due to the inherent viscoelastic behaviour of polymeric structures that comprise the adhesive; however, this is also what makes the adhesive and its mechanical behaviour so sensitive to loading rates.

Temperature fluctuations produce noticeable changes in the adhesive’s characteristics, which in turn provide noticeably altered adhesive joint behaviour. This impact of temperature on the strength of adhesive joints has been the subject of numerous investigations, and these studies state that testing at temperatures below room temperature leads to a decrease in joint toughness, while testing at higher temperatures leads to a decrease in joint strength.

Keeping the results in mind, there is no question that the tests performed at room temperature have the highest values of energy release rate. In other words, it can be observed that for the specimen tested at high temperatures, it does not have as much fracture toughness when submitted to certain load rates. This is probably because the temperature of 60 °C is too high bearing in mind the $T_{g}$ of −5 °C for this adhesive; therefore, the temperature in question will end up degrading the adhesive properties.

Regarding the low energy release rate values for the specimen tested at −30 °C, this may be a result of the phenomenon related to thermally induced physical changes that, at temperatures below the $T_{g}$ point, cause the adhesive becomes more brittle.

Thus, in a more general view, both temperature and strain rate influence the rate of energy release, and, in a way, both variables can work in symbiosis to alter the values in different ways, allowing a greater number of applications when combined.

**Figure 11:**
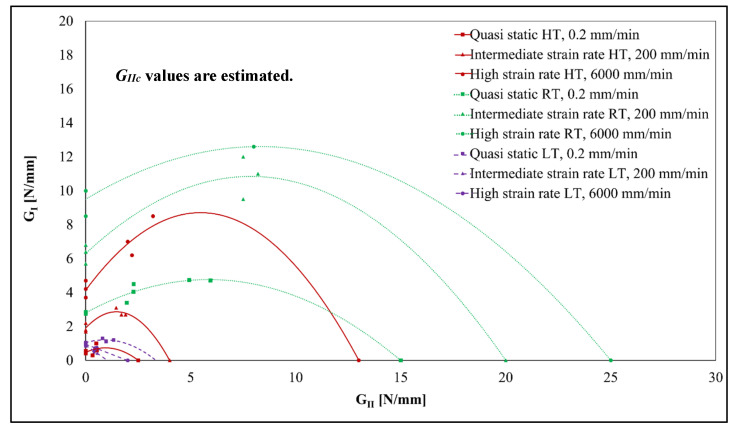
Fracture envelopes.

Figure 11 shows the fracture envelope for this adhesive. As can be seen in the figure, all the *G_IIc_* values have been estimated, because if a test were performed closer to pure mode II, with a mode-mixity higher than 45°, there would be a risk of plastic deformation of the substrate before crack propagation in the adhesive layer, especially under higher strain rate conditions at high and room temperature. The results of the quasi-static test at 23 °C, performed at a mixed-mode angle of 60 °C, were also taken into account to obtain the best possible approximation of the fracture envelope.

## 5. Conclusions

The study of the effects of strain rate and temperature on the mixed-mode fracture behaviour of the polyurethane adhesive has provided valuable insights into the mechanical properties and behaviour of adhesives under different loading conditions and service temperatures.

Thereby, the critical fracture energy for both mode I and mode II parts of the mixed mode of this polyurethane adhesive was analysed under three different loading rates (0.2 mm/min, 200 mm/min, and 6000 mm/min) and three temperatures (−30 °C, 23 °C, and 60 °C).

From the research carried out and the results obtained, the main conclusions drawn from this are the following:

- The critical strain energy release rate is greatly sensitive to the service temperature. The loading rate also greatly influences this parameter depending on the temperature to which the specimen is subjected.
- The maximum load supported by the specimen increases with increasing loading rate for temperatures above Tg.
- Under quasi-static conditions, the fracture energy increased around 3.4 times from below Tg to room temperature, and this disparity was exacerbated by an increase in loading rate.
- It was found that the influence of temperature is relatively more important for intermediate and high strain rates, as on the results at these two strain rates, GI increased by a factor of 35 for intermediate strain rate and 38 for high strain rate from low temperature (−30 °C) to room temperature (23 °C). GII also increased for the same conditions by factors of 25 and 95 times, respectively.
- Both strain rate and temperature exert a comparable influence on both mode I and mode II of the mixed mode, albeit mode I appears to exhibit an overall greater sensitivity to these effects.
- According to the results presented, the best adhesive performance was obtained for higher loading rates at room temperature with a GI of approximately 13 N/mm and GII of around 7 N/mm.

## Figures and Tables

**Table 1:** Physical and mechanical properties of the polyurethane adhesive at room temperature [15].

Property	Polyol	Isocyanate	Mixed
Specific gravity at 25 °C ($g/{\mathrm{cm}}^{3}$)	1.57	1.22	-
Viscosity at 25 °C (mPa.s)	7000	20	1100
$T_{g}\;$(°C)	−5 3.4 ± 0.09 33.4 ± 1.34 20.3 ± 1.23		
Maximum tensile strength (MPa)			
Maximum tensile strain (%)			
Young’s Modulus (MPa)			

**Table 2:** Summary of test conditions.

		Temperature		
		Low Temperature −30 °C	Room Temperature 23 °C	High Temperature 60 °C
**Loading Rate**	Quasi-static 0.2 mm/min	Mixed-mode test (45°)	Mixed-mode test (45°/60°)	Mixed-mode test (45°)
	Intermediate Speed 200 mm/min	Mixed-mode test (45°)	Mixed-mode test (45°)	Mixed-mode test (45°)
	High Speed 6000 mm/min	Mixed-mode test (45°)	Mixed-mode test (45°)	Mixed-mode test (45°)

## Data Availability

Not applicable.
